# Experimental Study on Glaze Icing Detection of 110 kV Composite Insulators Using Fiber Bragg Gratings

**DOI:** 10.3390/s20071834

**Published:** 2020-03-26

**Authors:** Jie Wei, Yanpeng Hao, Yuan Fu, Lin Yang, Jiulin Gan, Han Li

**Affiliations:** 1 School of Electric Power, South China University of Technology, Guangzhou 510640, China; epjie1123@mail.scut.edu.cn (J.W.); epyuanfu@mail.scut.edu.cn (Y.F.); eplyang@scut.edu.cn (L.Y.); 201630214199@mail.scut.edu.cn (H.L.); 2 State Key Laboratory of Luminescent Materials and Devices, South China University of Technology, Guangzhou 510640, China; msgan@scut.edu.cn

**Keywords:** Fiber Bragg Grating (FBG), composite insulator with embedded FBGs, glaze icing, icing detection

## Abstract

Icing detection of composite insulators is essential for the security and stability of power grids. As conventional methods have met difficulties in harsh weather, a 110 kV composite insulator with embedded Fiber Bragg Gratings (FBGs) was proposed for detecting glaze icing in this paper. FBG temperature compensation sensors in ceramic tubes were adopted for simultaneous measurement of icicle loads and temperature. Then, temperature calibration experiments and simulated icicle load experiments were carried out to obtain temperature and icicle load characteristics of FBGs. The results showed that temperature sensitivities of FBG strain sensors and FBG temperature compensation sensors were 18.16 pm/°C, and 13.18 pm/°C, respectively. Besides, wavelength shifts were linearly related to icicle loads within the polar angle range of −60° to 60°, and the load coefficient of FBG facing the icicle was -34.6 pm/N. In addition, the wavelength shift generated by several icicles was equal to the sum of wavelength shifts generated by each icicle within the polar angle range of −15° to 15°. Finally, icicles can cause wavelength shifts of FBGs within a big shed spacing. The paper provides a novel icing detection technology for composite insulators in transmission lines.

## 1. Introduction

Since the 21st century, the global climate has changed dramatically, and the shortage of conventional energy has become increasingly severe. It is significant to construct smart grids for the electric power industry [1]. Currently, the sensing technologies of electrical equipment are an essential foundation to ensure safe operation, promote controllability, and achieve intelligence of smart grid [2].

In March 2019, the Internet Department, State Grid Corporation of China, published an outline for the construction of the Ubiquitous Power Internet of Things (UPIoT). UPIoT includes four layers: sensing layer, network layer, platform layer, and application layer. In particular, the sensing layer is comprised of various sensors, edge computing equipment, and local communication networks to achieve acquisition, aggregation, and processing of data from conditions of power equipment. In summary, intelligent sensing technologies are one of the core foundations of the sensing layer in UPIoT [3].

Insulators in transmission lines are easily covered by ice in cold and high humidity regions, resulting in flashover accidents and even severe disasters of power grids. To date, there have been several methods (e.g., ultraviolet imaging, unmanned aerial vehicle (UAV), and video camera) to detect icing conditions on insulators. Wu and Liao [4,5] processed aerial insulators images from UAV based on a texture segmentation algorithm and a robust detection algorithm. On the other hand, some researchers monitored icing conditions of insulators and conductors using video cameras installed on power towers. Huang proposed an online technology for measuring icing shape on transmission lines based on vision and force sensors. The technology can accurately represent the icing shape on transmission lines [6]. Hao proposed a GrabCut segmentation algorithm and processed iced insulator images captured under artificial icing simulations and natural conditions. The results demonstrated that the method can recognize the icing conditions of in-service glass insulators [7]. The above technologies were helpful to improve performance of detecting iced insulators. However, in harsh environments, unmanned aerial vehicles are not able to fly long distances. In addition, cameras on power towers are easy to lose power supply and covered by ice causing blurry images. Consequently, the technologies are not suitable for long-term monitoring iced insulators in all weather. 

Currently, optical fiber sensors (OFS) have been widely used in various electrical equipment, such as transmission lines [8,9], power transformers [10,11], and insulator strings [12]. OFS is small in size, immune to electromagnetic interferences, chemically resistant, and has no power supply requirements. Besides, the composite insulator is long and stick-shaped. Therefore, OFS can be embedded into composite insulators, which establish distributed optical fiber sensing networks of the power grid. 

Fiber Bragg grating (FBG) is one of the typical OFS, and it has been successfully developed in monitoring conditions of composite insulators. In 1992, Seike et al. [13] first proposed an optical fiber composite insulator for detecting fault points in electric power transmission networks. An optical fiber was embedded in a central through-hole of the composite insulator. However, the insulation properties of the method were not satisfactory. Trouillet et al. [14] investigated strain distribution and internal temperature of a core rod using an optical fiber with six FBGs. The method provided the basis for detecting operating conditions of core rod in the composite insulator based on FBGs. Central China Power Group [15] and China Electric Power Research Institute [16,17,18,19,20,21,22] implanted FBGs into the core rod of composite insulators, and performed calibration tests to research the relationship between the center wavelength of FBG and the temperature/stress of composite insulators. From 2010 to 2019, the composite insulators with FBG embedded have been operated on 110 kV [21], 220 kV [17], 500 kV [17], and UHV [22] transmission lines, which validated the feasibility of the proposed method in the field. However, the methods embedded optical fibers into the core rod, which were not suitable for detecting glaze icing on the composite insulator sheds.

For theoretical analysis, Kumosa [23,24] and Portnov [25] established numerical models that can be used to evaluate the relationship between Bragg wavelength shift and internal stress of the core rod in composite insulators. Kerrouche et al. [26] calculated strain/shear stress from a substrate to FBGs. The results showed a relatively uniform stress distribution along the substrate. Chen et al. [27,28] simulated reflection spectrum of FBGs based on analysis of the stress distribution of a core rod in a composite insulator.

The above studies have validated the feasibility of embedding FBG sensors into the core rod of composite insulators. In addition to those studies, few studies have investigated glaze icing detection of composite insulator based on FBG, although glaze icing detection is one of the significant characteristics for fault detection in insulators.

In this study, a 110 kV composite insulator with embedded FBGs was designed to detect glaze icing. Firstly, the FBG sensing principle and the design of FBG temperature compensation sensors were described. Secondly, the FBG arrangement in the composite insulator was presented in detail. Then, temperature calibration experiments were performed, and FBG temperature characteristics errors were analyzed as well. The results indicate that the compensation method can measure temperature and icicle loads of FBGs simultaneously. Moreover, a polar coordinate system was proposed for describing positions of simulated loads and FBGs on insulator sheds. Finally, simulated icicle load experiments were performed to find the relationship between an icicle, several icicles, icicle positions, and wavelength shifts. The proposed method introduces a novel way for glaze icing detection of composite insulators in transmission lines.

## 2. Detection Principle

### 2.1. FBG Sensing Principle

An FBG is a spatial phase grating inscribing into the photosensitive fiber core. The FBG has a significant property of serving as a narrow band filter or reflector. More specifically, it can reflect a narrow band of light with a specific wavelength from an incident broad source, and the rest of the light with other wavelengths passes through the FBG. The FBG reflection wavelength is defined as λ_B_ [29]:

(1) $$\lambda_{B}=2n_{\mathrm{eff}}\cdot \Lambda$$

where *n*_eff_ is the effective refractive index of the optical fiber, and Λ denotes the periodicity of the grating.

Temperature, stain, or other environmental parameters would change the refractive index and periodicity of the FBG resulting in a wavelength shift. In this case, the FBG wavelength shift caused by temperature change (Δ*T*) and axial strain (*ε*) can be expressed as [30]:

(2) $$\begin{array}{ll} \Delta \lambda_{B} & =\lambda_{B}(\alpha_{f}+\xi )\Delta T+\lambda_{B}(1-P_{e})\varepsilon \\ & =K_{B,T}\Delta T+K_{B,\varepsilon }\varepsilon \end{array}$$

where *α_f_* denotes the thermal expansion coefficient, *ξ* is the thermo-optic coefficient, *P_e_* is the elastic-optic coefficient, and *K*_B,*T*_ and *K*_B,*ε*_ are both constant which denote the temperature sensitivity (pm/°C) and the axial strain sensitivity (pm/με), respectively.

### 2.2. Simultaneous Measurement of FBG Temperature and Strain

Glaze icing can change the temperature and strain of insulator sheds, which both cause the wavelength shifts of FBG at the same time. Therefore, it is necessary to propose a method for measuring FBG temperature and strain simultaneously. Specifically, two FBGs (i.e., FBG-1 and FBG-2) are inscribed in an optical fiber. FBG-1 is affected by temperature and strain, whose wavelength shift can be described as Equation (3). In contrast, FBG-2 is packaged by a designed housing that is not affected by strain. Thus, the wavelength shift of FBG-2 can be described as Equation (4).

(3) $$\Delta \lambda_{B1}=(K_{B1,T}\Delta T+K_{B1,\varepsilon }\varepsilon_{1})$$

(4) $$\Delta \lambda_{B2}=K_{B2,T}\Delta T$$

Therefore, the wavelength shift of FBG-1 caused by strain can be described as:

(5) $$\Delta \lambda_{compensation}=\Delta \lambda_{B1}-\frac{K_{B1,T}}{K_{B2,T}}\Delta \lambda_{B2}$$

## 3. Experiments

### 3.1. Fiber Bragg Grating

In this paper, FBGs with different reflection wavelengths have been inscribed in single mode optical fibers with polyimide coating using phase mask technology. The diameter, effective length and reflectivity of each FBG are about 250 μm, 10 mm and 90%, respectively. The initial wavelengths of FBGs in room temperature are within a range of 1510 to 1590 nm. The strain measurement of FBG ranges from −2500 to 2500 µε, and the accuracy rate is 1‰ F.S. Moreover, the operating temperature ranges from −40 to 120 °C. More specifically, three optical fibers were embedded into a composite insulator and labeled as 1#, 2#, and 3#, respectively.

**Figure 1:**
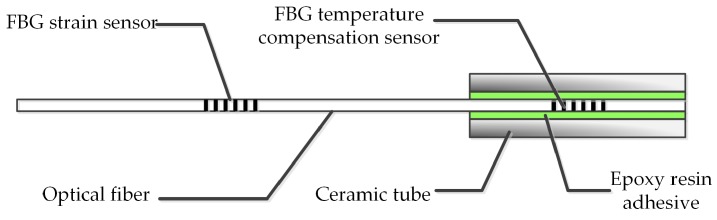
Fiber Bragg Grating (FBG) strain sensor and FBG temperature compensation sensor in an optical fiber.

In order to measure icicle loads and temperature simultaneously, the FBG temperature compensation sensor was packed in a ceramic tube, which is cylindrical, with a length of 30 mm and a diameter of about 2 mm (Figure 1). Besides, epoxy resin adhesive was injected into the ceramic tube for insulation. The FBG temperature compensation sensors are not affected by icicle loads due to the high elasticity modulus of the ceramic tube.

### 3.2. A 110 kV Composite Insulator with Embedded FBGs

**Figure 2:**
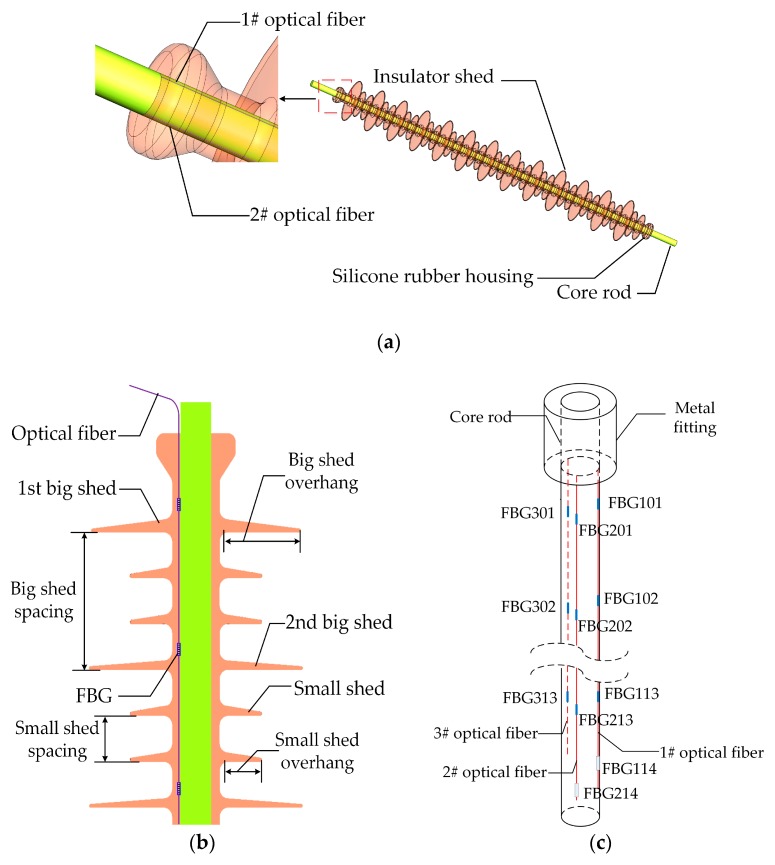
Diagram of a 110 kV composite insulator with embedded FBGs. (**a**) The parts of a composite insulator with embedded FBGs; (**b**) Relative positions between FBGs and insulator sheds; (**c**) Distribution diagram of three optical fibers with FBGs.

The type of 110 kV composite insulator is FXBW-110/100, whose parameters are listed in Table 1. The insulator has 13 big sheds, which are projected from the insulator trunk, and is intended to increase the creepage distance [31], as shown in Figure 2a.

Three optical fibers, 1#, 2#, and 3#, were embedded vertically into the composite insulator during silicone rubber injection process. Besides, the optical fibers were at a 120° interval around the central axis of the composite insulator (Figure 2). Fourteen FBGs were inscribed in 1# and 2# optical fiber respectively, and thirteen FBGs were inscribed in 3# optical fiber. The FBGs were labeled as FBG-mn, where m was the label of the optical fibers (i.e., 1, 2, and 3), and n was the label of the FBGs (i.e., 1, 2… and 14). For example, the fourteen FBGs in the 1# optical fiber were separately labeled as FBG101 - FBG114. Specifically, FBG101 - FBG113 were all FBG strain sensors, which were designed above each big shed root in silicone rubber housing (Figure 2b), and FBG114 was an FBG temperature compensation sensor. Similarly, FBG201 - FBG213 in 2# optical fiber and FBG301 - FBG313 in 3# optical fiber were all FBG strain sensors. In addition, FBG214 was also an FBG temperature compensation sensor.

After proper preparation of the core rod surface, three optical fibers with FBGs were put on the core rod surface according to the arrangement in Figure 2c. Then, two ends of optical fibers were fixed, and midpoints between two FBGs were adhered on the core rod using room temperature vulcanized silicone rubber (RTV) until curing. The core rod and end fittings were joined by a pressing process. Finally, the core rod and optical fibers were placed in a vacuum injection machine, and the pressure remained constant during the process of injecting silicone rubber and curing. 

### 3.3. The Sensing System

**Figure 3:**
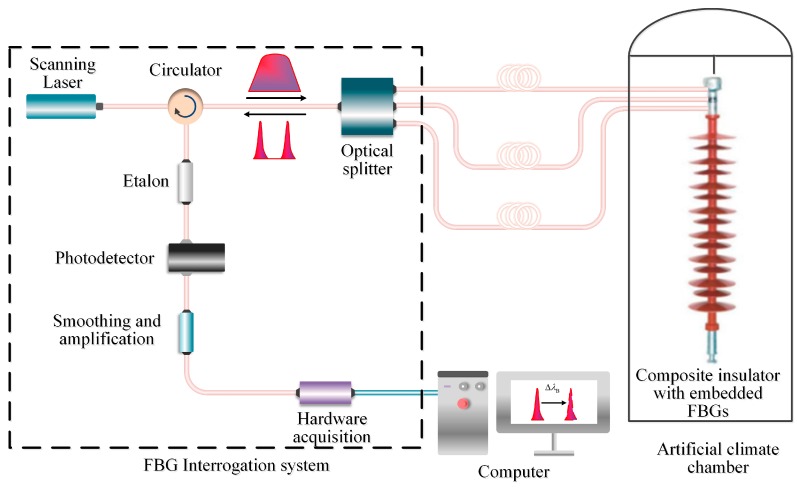
Sensing system of the composite insulator with embedded FBGs.

The sensing system was made up of three parts, i.e., the 110 kV composite insulator with embedded FBGs, an FBG interrogation system, and an artificial climate chamber, as depicted in Figure 3. The type of interrogation system is JEME-iFBG produced by Shenzhen JEMETECH Company in China. Specifically, the interrogation system can acquire signals of 6 channels simultaneously based on a high-power wavelength scanning laser, and each channel can detect 48 FBGs at most. With a wavelength range of 1510 to 1590 nm and a wavelength accuracy of 1 pm, the interrogation system can satisfy the requirement for detecting glaze icing on composite insulators. 

The artificial climate chamber was used to simulate the low-temperature environment during glaze ice accretion on composite insulators. Specifically, the artificial climate chamber offered a temperature change from −15 °C to room temperature (20 °C) with an accuracy of 1 °C. In experiments, three optical fibers were pulled out from the chamber, and the interrogation system was linked to the computer using the USB port for data recording, analysis, and transfer.

### 3.4. Temperature Calibration Experiments

Previous studies have demonstrated that glaze ice is easy to accrete on insulator sheds when the temperature is between −8 and 0 °C in the field [32]. Besides, according to IEEE Standard 1783-2009, the air temperature should remain between −15 and −5 °C for depositing artificial ice on insulator sheds in a climate chamber [33]. Thus, in the temperature calibration experiments, the air temperature in the climate chamber was set between −15 and +15 °C. The process was as follows:

(1) The 110 kV composite insulator with embedded FBGs was put into the artificial climate chamber. Three optical fibers were pulled out from the chamber, and connected with the demodulator and computer through outlet fibers. The wavelengths of all FBGs were recorded as initial wavelengths when the temperature was 0 °C.
(2) The temperature range of the artificial climate chamber was set from −15 to +15 °C, and the wavelength shifts were recorded at intervals of 3 °C. A previous research has indicated that the temperature of FBG in the composite insulator was equal to the temperature in the chamber after 8 hours [18]. Therefore, after being placed into the chamber for 8 h, the temperature of the composite insulator could be considered as a constant and recorded if a wavelength rate was less than 3 pm/min [18]. After averaging 10 sets of data, the results of temperature calibration experiments were created.

### 3.5. Simulated Icicle Load Experiments

**Figure 4:**
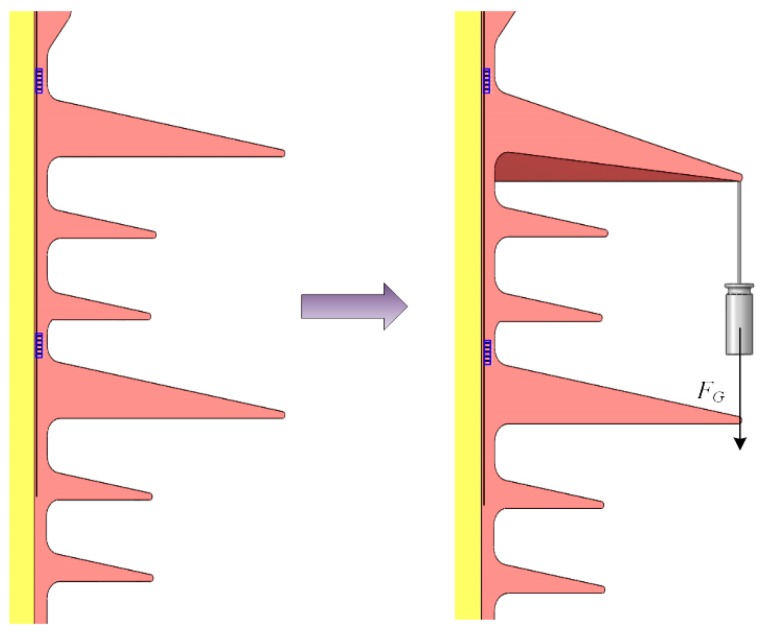
Hanging weights on a big shed for simulated loads of icicles [36].

Many studies have indicated that the common glaze ice accretion on insulators causes the highest probability of ice flashover occurrence [34,35]. Therefore, in order to find the relationship between glaze ice and wavelength shifts, weights were hung on the edge of a big shed for simulating glaze ice loads generated by icicles [36] (Figure 4). 

In order to describe icing conditions of composite insulators quantitatively, icicle length and icicle bridged degree were proposed, which can be described as [37,38,39]:

(6) $$\eta =\frac{S-d}{S}\times 100\%=\frac{L}{S}\times 100\%$$

where *η* denotes the bridged degree of a big shed spacing, *S* is the big shed spacing, *d* is the air gap distance, and *L* is the icicle length.

**Figure 5:**
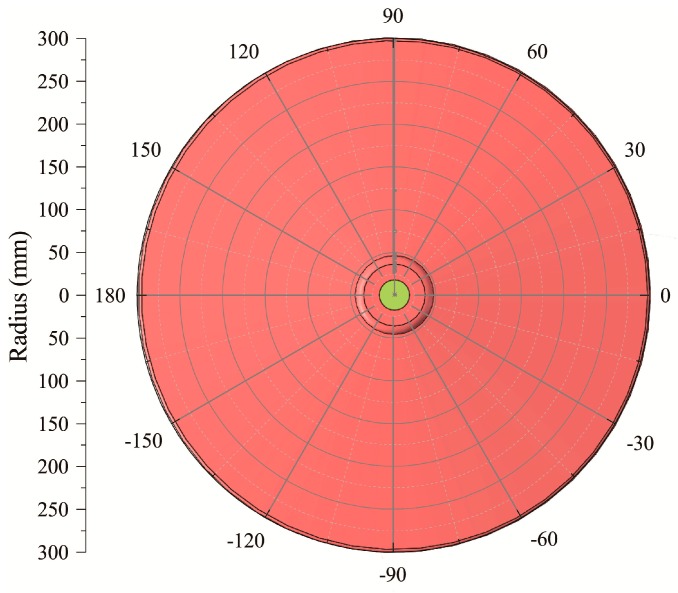
The polar coordinate system on insulator sheds.

In order to describe positions of simulated loads and FBGs on insulator sheds quantitatively, a polar coordinate system was proposed, where the midpoint of the core rod was the original point (Figure 5). The position was considered as (*r*, *θ*, *n*), where *r* denoted the radius (mm), *θ* denoted the polar angle (°), and *n* denoted the number of big sheds in the insulator. For example, the coordinates of FBG101, FBG201, and FBG301 were (9, 0°, 1), (9, 120°, 1), and (9, −120°, 1), respectively. When the simulated load was on the edge of the 1st big shed facing the 1# optical fiber, its coordinate was (61, 0°, 1). In this paper, the *r* of simulated loads were all 61 mm, so the coordinates of simulated loads could be simplified to *P*(*θ*, *n*).

The process was as follows:

(1) The 110 kV composite insulator with embedded FBG was placed in the artificial climate chamber, and the air temperature was set as -10 °C during the experiments. The optical fibers were pulled out from the chamber, and connected with the demodulator and computer.
(2) Geometric characteristics of an icicle on insulators were proposed in [37]. In this paper, the weights were set as 0.5, 1.0, 1.5, 2.0, and 2.5 N, corresponding to icicle lengths and bridged degrees, as shown in Table 2. The icicle lengths and bridged degrees are common parameters in icicle growth experiments [38,39].
(3) Weights were hung on insulator big sheds. The wavelength shifts were continuously recorded when wavelength rates were stable (< 3 pm/min) [18]. After averaging 10 sets of data, the results were created.

## 4. Results and Discussion

### 4.1. The Relationship between Wavelength Shifts of FBGs and Temperature

The wavelength shift mean deviation (WSMD) was proposed to discuss the temperature characteristics of FBG strain sensors in an optical fiber. For example, the WSMD of 13 FBG strain sensors in 1# optical fiber, i.e., FBG101 - FBG113, was expressed as *f*_1_, which can be defined as:

(7) $$f_{1}=\frac{\sum_{n=1}^{13}|\lambda_{n}-\bar{\lambda }|}{13}$$

where n denotes the label of FBG strain sensor in 1# optical fiber, *λ*_n_ denotes the wavelength shift of FBG1n at a temperature, and $\bar{\lambda }$ denotes the average value of wavelength shifts of 13 FBG strain sensors in 1# optical fiber at the temperature.

**Figure 6:**
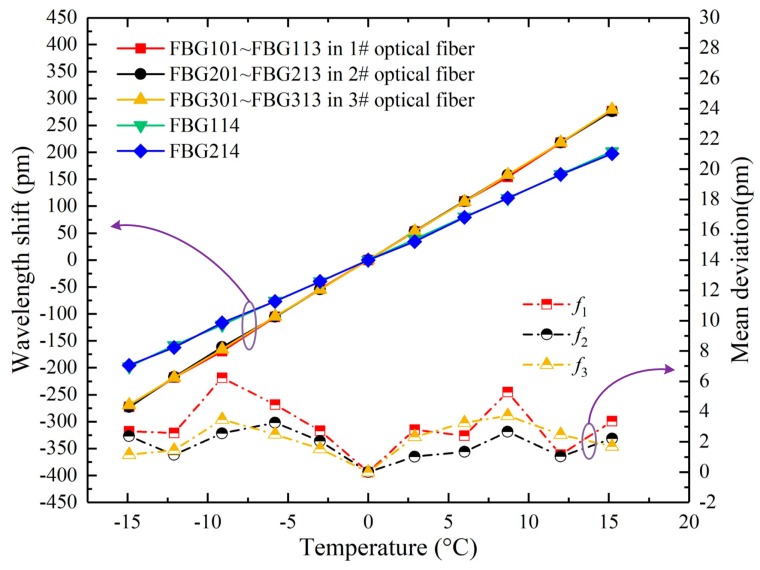
The relationship between wavelength shifts of FBGs and temperature.

Similarly, *f*_2_ and *f*_3_ represent the WSMD of FBG strain sensors in 2# and 3# optical fiber, respectively. As shown in the right Y axis of Figure 6, the mean deviations were all within 8 pm, which showed that wavelength shifts of FBG strain sensors in an optical fiber were seen as the same at a temperature.

Therefore, wavelength shifts dependent on temperatures of FBG101 - FBG113, FBG201 - FBG213, and FBG301 - FBG313 were shown in red, black, and yellow solid lines, respectively (Figure 6). The solid lines showed that wavelength shifts of all FBG strain sensors were linear with temperature, and goodness of fit *R*^2^ were all greater than 0.997. Therefore, the temperature sensitivities of all FBG strain sensors were approximately 18.16 pm/°C.

The green and blue lines showed that wavelength shifts of FBG114 and FBG214 were linear with temperature, and goodness of fit *R*^2^ were approximately 0.997. The temperature sensitivities of FBG temperature compensation sensors were 13.18 pm/°C. Therefore, according to Equation (5), the wavelength shift of an FBG strain sensor caused by strain was as follows:

(8) $$\begin{array}{ll} \Delta \lambda_{compensation} & =\Delta \lambda_{S}-\frac{18.16}{13.18}\Delta \lambda_{T}\\ & \approx \Delta \lambda_{S}-1.38\Delta \lambda_{T} \end{array}$$

where Δ*λ_S_* denoted the wavelength shift of an FBG strain sensor, and Δ*λ_T_* denoted the wavelength shift of an FBG temperature compensation sensor.

### 4.2. A Simulated Load of an Icicle on a Big Shed

**Figure 7:**
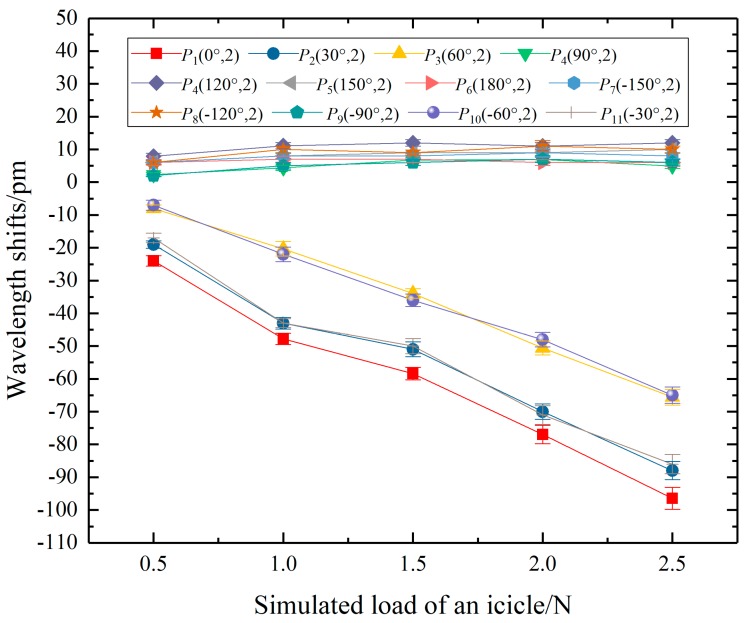
The relationship between the wavelength shift of FBG102 and simulated load generated on different positions.

The wavelength shift of FBG102 changed with the simulated load of one weight at *P*_1_(0°, 2), as the red line in Figure 7. When a simulated load on the edge of the 2nd big shed moved at a 30° interval, the wavelength shifts of the FBG102 were shown in Figure 7.

It was found that the wavelength shifts of FBG102 were linearly related to the simulated load of one weight at *P*_1_(0°, 2), *P*_2_(30°, 2), *P*_3_(60°, 2), *P*_10_(−60°, 2), and *P*_11_(−30°, 2), respectively, corresponding to a polar angle range of -60° to 60°. The linear fitting formulas were described as:

(9) $$\Delta \lambda_{1}=-34.6F_{1}-8.9,\;R^{2}=0.990$$

(10) $$\Delta \lambda_{2}=-33F_{2}-4.7,\;R^{2}=0.984$$

(11) $$\Delta \lambda_{3}=-28.8F_{3}+7.8,\;R^{2}=0.997$$

(12) $$\Delta \lambda_{10}=-28.4F_{10}+7,\;R^{2}=0.998$$

(13) $$\Delta \lambda_{11}=-33.2F_{11}-3.6,\;R^{2}=0.978$$

where *F_i_|_i_*_= 1,2,3,10,11_ were the simulated load generated at *P_i_|_i_*_= 1,2,3,10,11_, Δ*λ_i_|_i_*_= 1,2,3,10,11_ were the wavelength shifts of the FBG102, respectively.

According to the above formulas, the absolute value of the load coefficient in Equation (9) was the largest (i.e., 34.6 pm/N). Therefore, the load coefficient of FBG facing the load was the highest. Besides, with the increase of the polar angle of the load, the absolute value of the load coefficient gradually decreased.

Comparing the Equation (10) with Equation (13), Equation (11) with Equation (12), the load coefficients of FBG102 were approximate when the loads were symmetrical about the polar angle of 0°. It can be concluded that the wavelength shifts of FBG were equal when two loads were symmetrical about the extension line from the original point to the FBG.

In contrast, when the polar angles of icicle load were from 90° to −90°, with the increase of the icicle load, the wavelength shifts appeared to remain stable. The reason for this was that FBG102 was located 86.27 mm away from *P*_4_ and *P*_9_, even 140 mm away from *P*_6_. The strain transferring to FBG102 was so small that the wavelength shifts were all less than 12 pm.

### 4.3. Several Simulated Loads of Several Icicles on A Big Shed

**Figure 8:**
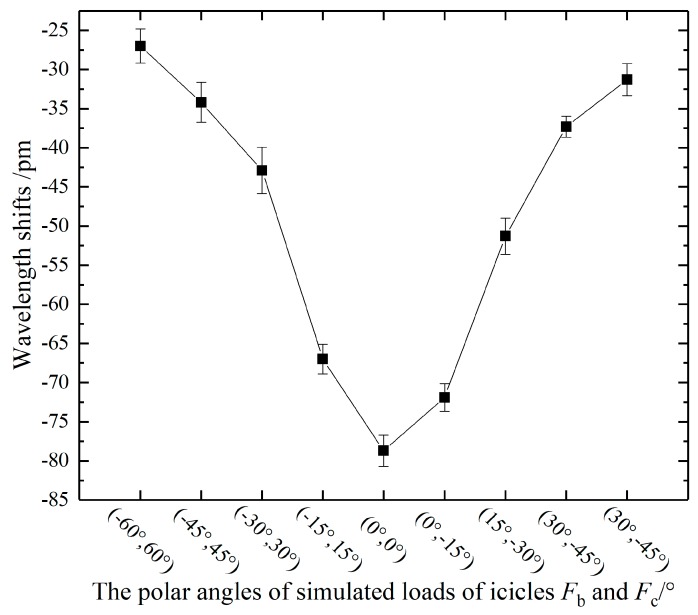
The relationship between the wavelength shift of FBG103 and the positions of simulated loads.

In order to simulate several icicles accretion on different positions of an insulator shed, three simulated loads were hung on a big shed in the insulator. It is clear from Section 4.2 that wavelength shifts were easily affected by the icicle load with polar angle ranges of −60° to 60°. Therefore, a simulated load *F*_a_ of 1 N was at *P*_a_(0°, 3), and the other two simulated loads *F*_b_ and *F*_c_ of 0.5 N were at (*θ*°, 3) where *θ* changed in a polar angle range of -60° to 60°. The relationship between the wavelength shifts and the changing polar angles (*θ*_b_, *θ*_c_) were shown in Figure 8.

It was found that the absolute value of wavelength shift decreases with the distance from *P*_a_(0°, 3) to load point (e.g., *F*_b_ and *F*_c_). In addition, when the polar angle of *F*_b_ and *F*_c_ were between -15° and 15°, the wavelength shifts of FBG103 were between 67 and 79 pm. In contrast, the wavelength shift generated by the sum of *F*_a_, *F*_b_, and *F*_c_ at 0° was 79 pm. The deviations of the wavelength shifts were within 12 pm. In view of the experimental error, it can be found that when the polar angle of *F*_b_ and *F*_c_ were between −15° to 15°, the wavelength shifts were similar to that generated by the sum of *F*_a_, *F*_b_, and *F*_c_ in 0°.

In conclusion, when the polar angles are between −15° and 15°, the wavelength shift generated by several icicles was equal to the sum of wavelength shifts generated by each icicle. In contrast, when the polar angles are between ±15° and ±60°, the absolute values of wavelength shifts would decrease with the distance from icicle positions to *P*_a_.

### 4.4. Wavelength Shifts of All FBGs in an Optical Fiber

To analyze wavelength shifts of all FBGs in an optical fiber when an icicle was generated on a big shed, a simulated load of 2 N was generated at (0°, 1), (120°, 1), and (−120°, 1), respectively.

**Figure 9:**
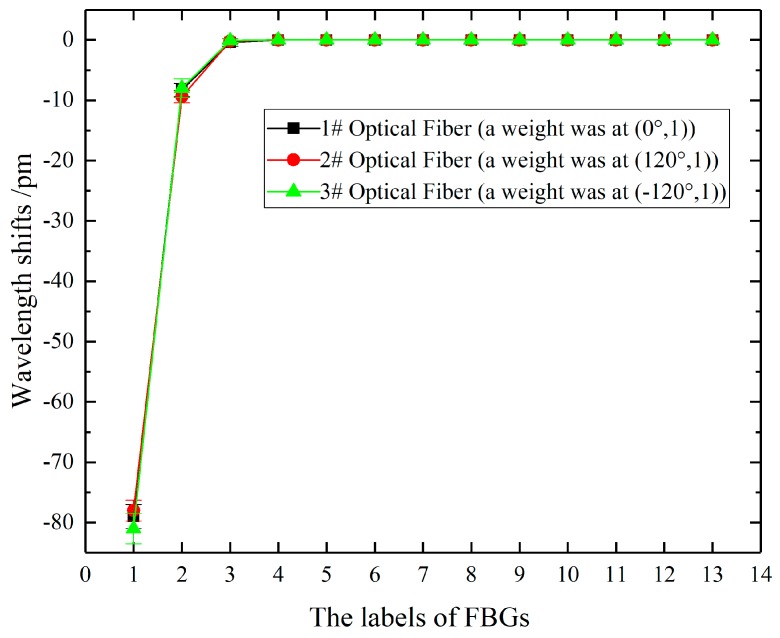
The wavelength shifts of FBG1n, FBG2n and FBG3n when a simulated load of 2 N was at (0°, 1), (120°, 1) and (−120°, 1), respectively.

It was found from Figure 9 that the wavelength shifts of the FBGm01 were -80 pm, those of the FBGm02 were less than −10 pm, while those of FBGmn (3 ≤ n ≤ 11) were almost 0 pm. The reason for this is that the silicone rubber is soft material, whose elasticity modulus is 2.6 MPa. The deformation of the 1st big shed was large under high loads, meanwhile, the strain transferring to FBGm02 was large enough to detect the wavelength shifts. As for FBGmn (3 ≤ n ≤ 11), the transferred strain was too small to change the wavelength shifts. In conclusion, icicle loads can cause wavelength shifts of the FBGs within one big shed spacing.

## 5. Conclusions

In this paper, a detection method for glaze icing of 110 kV composite insulators has been proposed. The 110 kV composite insulator with embedded FBGs is helpful to sense glaze icing conditions in all weather, offering much better versatility than conventional approaches. Three optical fibers with FBGs were embedded symmetrically into a 110 kV composite insulator. Besides, the FBG temperature compensation sensors were packaged in ceramic tubes so as to solve the problem of icicle loads and temperature cross-sensitivity. Then, temperature calibration experiments and simulated icicle load experiments were performed.

The results showed that temperature sensitivities of FBG strain sensors and FBG temperature compensation sensors were 18.16 pm/°C, and 13.18 pm/°C, respectively. FBG wavelength shifts were linearly related to the icicle loads within the polar angle range of −60° to 60°. In particular, the load coefficient of FBG facing the icicle was −34.6 pm/N. Moreover, within the polar angle range of −15° to 15°, the wavelength shift generated by several icicles was equal to the sum of wavelength shifts generated by each icicle. Finally, icicle loads can cause wavelength shifts of the FBGs within one big-shed spacing. Therefore, the icicle loads of composite insulators can be detected using FBGs in the laboratory.

In further research, a mathematical model and a location algorithm will be proposed for monitoring multiple icicle loads and positions simultaneously on the composite insulator. In addition, uncontrollable outdoor factors (e.g., sunlight, wind, rain, etc.) should be taken into considerations as well.

## Figures and Tables

**Table 1:** Parameters of a composite insulator FXBW-110/100.

Description		Values
Structural height		1240 mm
Insulation height		1036 mm
Leakage distance		4953 mm
Diameter of the core rod		18 mm
The big insulator shed	Shed overhang	61 mm
	Shed spacing	75 mm
	Number	13
The small insulator shed	Shed overhang	37 mm
	Shed spacing	25 mm
	Number	26

**Table 2:** Icicle load corresponds to the icicle length and icicle bridged degree with density of 0.9 g/cm^3^ [36,37].

Weights	Corresponding to the Icicle Length *L*	Corresponding to the Icicle Bridged Degree *η*
0.5 N	11 mm	15%
1.0 N	22 mm	29%
1.5 N	33 mm	44%
2.0 N	44 mm	59%
2.5 N	55 mm	73%
